# Everyday life experiences of Chinese community-dwelling oldest old who live alone at home

**DOI:** 10.1080/17482631.2023.2253937

**Published:** 2023-09-05

**Authors:** Jiayin Ruan, Weina Zheng, Yiyu Zhuang

**Affiliations:** a School of Nursing, The Hong Kong Polytechnic University, Hung Hom, Kowloon, Hong Kong Special Administrative Region, China; b Department of Nursing, Sir Run Run Shaw Hospital, Zhejiang University School of Medicine, Hangzhou, Zhejiang, China

**Keywords:** Aging, ageism, experiences, living alone, qualitative research

## Abstract

**Purpose:**

Older adults aged ≥ 80 years living alone at home are more likely to experience challenges. Daily life experiences regarding living alone are still limited in the Asian context. This study explored the everyday life experiences of older Chinese residents living alone at home. Research questions included: (1) How do Chinese community-dwelling old people describe everyday life experiences related to living alone? (2) What kind of difficulties and needs do Chinese community-dwelling older people living alone face in everyday lives? (3) How do Chinese community-dwelling older people cope with challenges faced in their everyday lives related to living alone?

**Methods:**

This was qualitative descriptive research. Purposive sampling was adopted to recruit 13 participants aged 80–92 years of age from communities and one hospital. Semi-structured interviews were conducted to collect data which was analysed by conventional content analysis.

**Results:**

Three themes were identified: theme 1–difficulty in finding a sense of belonging, theme 2–striving to maintain independence, theme 3–hard to gain a sense of control.

**Conclusions:**

This study provided novel insights into understanding the difficulties and needs of Chinese older people living alone at home. Three key challenges and associated strategies they used to cope with in daily lives were presented.

## Introduction

Ageing of the population is a global phenomenon. The number of people 80 years and older is expected to increase from 143 million in 2019 to 426 million in 2050 (United Nations, Department of Economic and Social Affairs, Population Division, 2020). Chronological age could be classified as youngest (60–69 years old), middle (70–79 years old), and oldest-old (≥80 years old) (Kydd et al., 2020). As people age, there are a variety of psychological, biological, and social changes in life. Compared to those in the first two age groups, individuals at an advanced age are more likely to experience obvious ageing symptoms, overall health decline and multiple comorbidities, falls and fall-related injuries, and losses (Jaul & Barron, 2017; Wang et al., 2020). Staying at home, the primary manifestation of ageing in place, is the strong preference of many older adults to spend later life (Han et al., 2020; Molina-Mula et al., 2020). Those in their 80s and older living on their own is no exception (Joint Center for Housing Studies of Harvard University, 2019).

However, due to advanced age, more obvious comorbid symptoms and living alone, those older groups may face more difficulties in daily life compared to home-dwelling sexagenarians or septuagenarians who also live alone. This raises several questions: what difficulties do those aged 80 years or older living at home alone confront? How do they deal with these challenges? What needs do they require? The current study seeks to provide answers to these questions by exploring the daily lives of the oldest population who live alone at home using qualitative research methods. Revealing the everyday life experiences related to living alone helps the family members of older adults better understand this population, guides healthcare professionals to evaluate and provide community services based on their needs and preferences, and also promotes the development of future targeted interventions.

Currently, qualitative studies from Western countries have explored the everyday life experiences related to living alone of home-dwelling oldest old people and generated rich findings. Specifically, different types of risk (Bedin et al., 2019), feeling insufficient in daily life (Ness et al., 2014a, 2014b), loneliness becoming more and more intractable (Lacatena & Sommantico, 2022), modifying daily living patterns to adjust to different energy resources (Hinck, 2004), valuing daily rituals (Nicholson et al., 2013), and supportive relationships (Paine et al., 2022).

On the contrary, Chinese researchers mainly paid attention to issues related to the widening urban-rural disparities in disability and longevity (Liu et al., 2019), quality of life differences based on geographic variations (Gu et al., 2019), and nutritional status (Lai et al., 2021) of the oldest population using quantitative studies. However, there remains limited research exploring daily life experiences related to living alone of older people living in the community in the Chinese context.

In qualitative research, reality, such as everyday life experiences related to living alone, is constructed within cultural, historical, and socioeconomic contexts (Korstjens & Moser, 2017). Due to the differences in cultural background, socioeconomic status and pension systems between China and western countries, it would be better to focus on the daily lives of the oldest people who live alone directly within the Chinese context.

Thus, this study aims to explore the everyday life experiences of Chinese community-dwelling oldest old individuals who live alone at home, reveal their difficulties and needs, and describe how they cope with challenges in their solitary life. We, therefore, asked the three related research questions as follows: (1) How do the oldest old Chinese community-dwelling describe their everyday life experiences related to living alone? (2) What kind of difficulties and needs do Chinese community-dwelling oldest old living alone face in their everyday lives? and (3) How do Chinese community-dwelling oldest old cope with challenges faced in their daily lives related to living alone?

## Materials and methods

### Study design

Descriptive qualitative research was adopted. This method has the essential characteristics of naturalistic enquiry (e.g., no manipulation or pre-selected study variables) (Lincoln & Guba, 1985), focusses on discovering what, who, and where of experiences or events, and aims to offer a straight descriptive summary of an event or experiences organized in a way that “fits” the data best (Sandelowski, 2000).

### Recruitment

Purposive sampling was used to recruit the oldest old people living alone at home in nine communities in two major cities (Hangzhou and Shaoxing) in Zhejiang Province, Southeast China, and one tertiary teaching hospital in Hangzhou. The inclusion criteria were individuals: (1) 80 years old and above; (2) living alone at home; (3) without hearing impairments; and (4) able to understand and speak Mandarin Chinese or either the Hangzhou or the Shaoxing dialect. The exclusion criteria included those who were (1) unable to participate in a face-to-face qualitative interview; (2) those living in long-term care facilities; and (3) individuals with a severe incapacitating or life-threatening disease.

### Data collection

Face-to-face, audio-recorded, semi-structured interviews were conducted from April 2021 to May 2022. An open-ended interview guide was developed based on the literature on the daily lives of very old people living alone in their own homes (Bedin et al., 2019; Hinck, 2004; Ness et al., 2014a, 2014b; Nicholson et al., 2013) and the previous experience of the researchers interacting with the oldest old people (Table I).

**Table I.** An interview guide.

Interview questions
1.Please describe an ordinary day in your life.
2.Please describe particular days in your life.
3.Please describe challenges you confront while living alone at home.
4.Please describe how you deal with challenges while living alone at home.
5.Please share your positive experiences while living alone at home.
6.Please share your negative experiences while living alone at home.

The question “please describe an ordinary day in your life” was often asked first. The follow-up questions were then used to clarify or explore the responses of the participants. For example, a participant said she used to enjoy playing *Mahjong* with other older adults, but was now unwilling to do so. JR further asked, “Please tell me more about different experiences of playing *Mahjong* with older people, both now and previously.” Participants were encouraged to speak freely. While the participant narrated, the interviewer actively listened, observed, and maintained neutrality. Field notes were used to record details of the interview settings and non-verbal information.

Qualitative interviews were conducted by JR, who has some experience in qualitative interviews, or by WZ, who is a novice in qualitative interviews in different interview settings (Table II). Each interview was transcribed verbatim. The interview time ranged from 23 to 163 minutes (*M* = 74.46).

**Table II.** Interview settings.

Location	N
Participants’ homes	11
First floor of the participant’s apartment	1
Demonstration classroom in a hospital	1

### Interview participants

Thirteen participants (2 men and 11 women) with a mean age of 85.31 years were included. All were Han Chinese. Twelve participants had medical insurance and owned the house where they lived. The demographic characteristics of the participants are presented in Table III.

**Table III.** Demographic characteristics of the participants (*n* = 13).

No.	Gender	Age	Education level	Occupation (previous)	Religion status	Marital status	Number of children	Place of children’ residence	Family relationship	Time of living alone (years)	Home ownership	Monthly personal income (CNY)	Medical insurance	Medical history (years)	Mobility aid usage
1	Woman	80	Secondary	Weaver	Christian	Widowed	3	Some in the same city	Healthy	7	Individual ownership	3800	Yes	Rheumatoid arthritis (18)	Cane
2	Woman	91	Illiterate	Weaver	Christian	Widowed	5	Some in the same city	Unhealthy	25	Individual ownership	5000	Yes	Hypertension (41), diabetes (19), gallstones (39)	Wheelchair
3	Woman	88	Secondary	Spinner	Buddhist	Widowed	3	All in the same city	Healthy	23	Individual ownership	4300	Yes	Diabetes (12)	Cane
4	Woman	85	Illiterate	Farmer	Christian	Widowed	4	All in the same city	Unhealthy	11	Rent	2000	Yes	Gastric ulcer (8), premature atrial contractions (1)	Independence
5	Woman	85	Primary	Knitter	Christian	Widowed	3	All in the same city	Healthy	2	Individual ownership	4000	Yes	Diabetes (5)	Cane
6	Man	80	Primary	Boatman	Buddhist	Widowed	3	All in the same city	Healthy	5	Individual ownership	2900	Yes	Diabetes (12)	Independence
7	Woman	80	Illiterate	Lathe man	Buddhist	Widowed	5	All in the same city	Healthy	0.6	Individual ownership	2010	Yes	Recurrent oral aphthous ulcers (5)	Independence
8	Man	86	Illiterate	Farmer	No religion	Single	0	Not applicable	General	36	Individual ownership	720	No	Cervical spondylosis (10)	Independence
9	Woman	92	Illiterate	Housewife	No religion	Widowed	4	All in the same city	Healthy	30	Individual ownership	1100	Yes	Hypertension (21), diabetes (4)	Independence
10	Woman	84	Illiterate	Farmer	Buddhist	Widowed	2	All in the same city	Healthy	3	Individual ownership	3000	Yes	Hypertension (30), atrial fibrillation (2)	Independence
11	Woman	86	Illiterate	Farmer	Buddhist	Widowed	3	All in the same city	Healthy	6	Individual ownership	5000	Yes	Hypertension (10), coronary artery disease (4), gout (1)	Independence
12	Woman	85	Illiterate	Farmer	Buddhist	Widowed	3	All in the same city	Healthy	5	Individual ownership	3300	Yes	Hypertension (6), diabetes (20)	Independence
13	Woman	87	Tertiary	Engineer	No religion	Widowed	4	All in the same city	Healthy	10	Individual ownership	4500	Yes	Hypertension (30), atrial fibrillation (0.58)	Independence

*Note*. CNY = Chinese Yuan Renminbi.

### Data analysis and rigor

Data analysis occurred simultaneously with data collection in the qualitative study. Conventional content analysis (Erlingsson & Brysiewicz, 2017; Hsieh & Shannon, 2005) was used to analyse the data by JR, who has experience conducting and reporting qualitative descriptive studies. On completion of the interview transcription, the coder reviewed the data by reading and rereading the interview transcripts multiple times to obtain a sense of the general understanding of the everyday life experiences of the oldest old who lives alone at home. Then, the coder, keeping the research aims and questions clearly in focus, divided the text into smaller parts (also known as meaning units) and condensed these meaning units through manual line-by-line coding. During this process, the coder retained the core meaning of the meaning units. For example, the meaning unit of “I often sit alone at home and feel very lonely” was coded into a condensed meaning unit of “feeling lonely at home alone.” Next, JR developed codes with an ability to describe the condensed meaning units by adjusting, re-considering, and re-coding, and grouped these codes into categories. For instance, the condensed meaning unit of “memory decline significantly” and “getting tired so easily” were condensed further into a relatively higher abstract level code as “noticeable ageing body.” Subsequently, JR compared these categories according to shared characteristics to determine which categories seemed appropriate to merge, thus forming a subtheme or a theme. For example, the categories of “maintaining financial independence,” “buying things with the help of mobility aids,” “technology-assisted purchasing” and “making full use of energetic time for doing necessary daily activities” were coded into a higher abstract level subtheme as “trying to meet basic living needs,” When “information redundancy” was yielded from the data (after the 10^th^ interview in the current study) (Braun & Clarke, 2021), saturation was achieved. A further interviews were conducted to further confirm saturation. The resulting thematic structure was reviewed by the research team.

Rigor was maintained through several strategies (Bradshaw et al., 2017; Milne & Oberle, 2005). Credibility and authenticity were ensured by establishing and maintaining good relationships with participants and verifying the accuracy of the interview transcripts by the researchers. JR has been acquainted with four of the participants for approximately 25 years, and both researchers have established mutual respect and good relationships with all participants. For criticality and integrity, we maintained reflective journals consistently to help us maintain sensitivity and see the everyday life experiences related to living alone through fresh eyes. For example, we noted how our roles (JR, young female with experience of interacting with the oldest old; WZ, young female clinical nurse) could influence data collection and interpretation.

### Ethical consideration

Ethical approval was granted from the Institutional Ethical Committee of Sir Run Run Shaw Hospital, Zhejiang University School of Medicine (No. 20201231–45). Written informed consent was obtained prior to each interview and each participant could leave the study at any time without providing any explanation. Prior to the study, the researchers recognized and acknowledged that there existed emotional potential for both participants and researchers. The negative emotions raised during the interview could help researchers engage with the lives of the participants and improve understanding of the study objectives. During the interview, the researcher ensures the comfort of the participant was a priority, provided empathic and understanding approach, maintained eye contact, listened carefully, was particularly mindful of the participant’s negative emotions, and ensured that the interviewee had calmed down after the interview was over. Furthermore, the researchers’ contact information was provided to the participants in case of inconvenient experiences or need of support. Names and other identifiers were removed from the interview transcriptions to ensure anonymity and to protect the participants’ confidentiality.

## Results

Three themes and eight sub-themes were identified to describe the daily life experiences of Chinese community-dwelling oldest old who live alone at home (Table IV).

**Table IV.** Daily life experiences of Chinese community-dwelling oldest old who live alone at home.

Themes	Subthemes
Difficulty in finding a sense of belonging	Home like a lonely island
	That is the home of my children
	Bullying by others
Striving to maintain independence	Trying to meet basic living needs
	Worries left untold
	Supported by spiritual strength
Hard to gain a sense of control	Body in continuous decline
	Ambiguous death

### Theme 1: difficulty in finding a sense of belonging

Achieving a sense of belonging relates to effective human interactions and can lessen loneliness. However, for community-dwelling oldest old who live alone at home, this can be difficult.

#### Home like a lonely island

Home was the place where the oldest old living alone spent most of his life. Due to the lack of interpersonal interaction (e.g., no regular visits from family or friends), home felt like “a lonely island”. Many participants, especially female participants, mentioned that they had a strong sense of loneliness most of the time. This feeling was especially evident during the period following the death of their spouses, when darkness fell, when the door closed once they got home and saw the empty house, when they were stricken by disease, or when an event suddenly occurred, but no one was around to help. *“Since my husband passed away, the house was deserted immediately. I felt so lonely, particularly when the sky became dark and I was alone in the apartment!”* (Participant 7)

One participant experiencing a sudden flare-up of rheumatoid arthritis described, *“The day before yesterday, I could not even get out of my bed, not to mention go to the toilet to pass urine. I was incredibly lonely, helpless, and mentally exhausted!” (Participant 1).*

Compared to those of similar age but were living with their spouses, some participants expressed admiration for older adults who had companions. Consequently, their sense of loneliness became more severe. *“I highly admire the old couple who lived downstairs … only because the husband and wife are both alive and can chat with each other every day.” (Participant 3)*

#### That is the home of my children

In the eyes of many participants, children’s homes belong to the children’s rather than their own, and their children have their own careers and lifestyle. If they do live together, the freedom of maintaining and practicing their familiar lifestyle (e.g., waking up early in the morning and going to sleep early in the evening) and preferred dietary habits (e.g., cooking longer to make food easier to chew) might be reduced or even lost entirely. *“I wouldn’t say that I liked to live with them (children) and eat the food they cooked, and vice versa…If living in their house, practicing my dietary habit might be hindered.” (Participant 4)*

Some participants chose not to live with their children because of the need to speak carefully to avoid conflict while talking with children, or their intuitions of being disrespected if living together for a long time. *“Living with my son and his wife (the daughter-in-law) is unpleasant. I lived alone. The daughter-in-law differs from my son, whom I can speak to casually. I have to talk to her politely and carefully; otherwise, it may destroy the family relationship if something unpleasant is said.” (Participant 13)*

#### Bullying by others

From the oldest old’s view, going out to places where older adults usually gather (e.g., parks, senior citizen activity centres, banks, and churches) could reduce loneliness, help them feel more involved and connected to others of the society. However, some participants indicated that they were found insufferable by younger adults playing *mahjong*/cards or chatting together due to their relatively steeper decline in physical function and cognitive performance. *“Those older adults slightly younger than me disliked playing cards with me because I had slower responses than them. They teased me like this ‘Oops. You played cards so badly!’ Since then, I have stopped playing with them.” (Participant 3)*

Some participants mentioned their own or their neighbours’ experiences of bullying or described similar news in the media. *“Most nannies treated older adults badly, especially those old people with long-term immobility problems. I heard that some even beat older people if they were not obedient.” (Participant 6). “You may have a bad experience in a nursing home. You never know.” (Participant 11)*

### Theme 2: striving to maintain independence

Independence was ultimately related to self-respect. Despite reaching an advanced age and living alone, the participants still attempted to maintain their independence.

#### Trying to meet basic living needs

For older people who lived alone at home, satisfying basic living needs was regarded the most fundamental part of their life. Financial independence was the critical step to achieving this. For some participantswere unfilial and did not show qualities expected of a good son or daughte, they maintained property and money instead of selling their house or giving monthly pensions to children. Otherwise, they will lose their right to economic freedom and would require requesting money based on the disposition of the children or their financial ability. *“You should have a pension and your own savings…When money is urgently needed, I can deal with it by myself. What could you do if you ask your children to pay for something if they do not have the money?” (Participant 2)*

With disposable earnings, some older adults with mobility issues were slowly able to go to the market to buy something using a cane or a wheelchair. Several older people with relatively higher education levels and willingness to use technology learnt to purchase things through e-commerce shopping platforms, such as *“Pinduoduo”* or *“Meituan.” I did my grocery shopping at “Duo Duo Grocery”. It was convenient, and no matter how much you bought ten yuan or twenty yuan, these groceries could be delivered and placed in a specific area near my house for me to pick up.” (Participant 1)*

Some participants, especially those who were older and/or those who had multiple chronic diseases, mentioned that they easily fatigued and had low energy. Therefore, they made full use of the mornings, which was when they felt fresh and energetic, to do the things necessary for their entire day, such as preparing food for meals. *“I was energetic in the morning. So, I prepared the fresh ingredients for a meat dish and a vegetable dish when I got up, and then cooked at noon.” (Participant 3)*

#### Worries left untold

This subtheme reflects an essential strategy for the oldest old people with the ability to self-care to maintain their solitary life independently. They knew that their situation of living alone could change and that their own lifestyle could be hampered if their children received any “bad news”, even those considered not urgent or unserious. This included feeling uncomfortable, feeling lonely, and experiencing slight missteps or non-severe falls. These potential changes were unacceptable when participants knew that they could handle the situation alone. Therefore, they hid bad news from family members. *“I have never told my son or his wife that I often feel lonely. If I tell them, this may make them in a pickle. They might want to solve this problem by inviting me to live with them, but I do not want to.” (Participant 3)*

Some participants whose children have migrated and settled overseas for many years opted not to inform their children about bad news, because they thought that this sharing could not bring any practical benefits but could make their children worry or even blame them, which will make them feel like a troublemaker. *“My principle of communicating with children was ‘reporting only what is good while concealing what is unpleasant.’ My daughter has settled in the USA for many years. Sharing bad news with her is not only meaningless due to her inability to provide practical help but will put a psychological burden on her.” (Participant 1)*

#### Supported by spiritual strength

For the oldest old population, living alone was full of challenges compared to the experiences of older adults younger than them. When encountering difficulties, some participants mentioned that religious beliefs gave them spiritual support and helped them overcome these adversities. *“I felt uncomfortable and vomited that day. No one was around. I just prayed to Jesus and later improved. God is our ever-present help in times of need!” (Participant 4)*

One participant stated that the fact that the children had been achieving excellently provided her with psychological comfort. *“Although I was disturbed by the disease now and then, I had psychological comfort when I thought that my three grandsons were doing well in America.” (Participant 1)*

### Theme 3: hard to gain a sense of control

Most very old individuals who lived alone struggled to maintain independence and tried to get a sense of control over life. However, when faced with a continuously declining body and unknown death at the end of life, they understood that little could ultimately be done.

#### Body in continuous decline

When people reached the age of 80 years and older, they felt that their physical function declined much faster. The main manifestations included memory loss, vision impairment, lack of energy, susceptibility to fractures, and unbalanced walking.

Despite these significant changes, some participants indicated that it was so terrible to be old and were disgusted with themselves. This sense of self-loathing was more apparent when compared to their relatively younger selves (e.g., their selves five or ten years prior) or to their capable selves at a younger age. *“I had a good memory and was good at doing sums in my head…However, now I cannot calculate accurately. This change definitely influences my mood.” (Participant 3)*

Despite being very careful, unpredictable fractures and slow recovery afterwards made participants realize that their physical decline was much worse and more uncontrollable than they thought. *“My left leg was nipped by a bus door and broke. Several years have passed since the fracture treatment. However, I still have a limp and need to walk with a cane.” (Participant 5)*

#### Ambiguous death

Death seems to be closer than ever before when individuals reach very old age. Most of the participants thought that their longevity was long enough and did not fear death itself, instead worrying about how they would die. “A quick death is a blessing” was expressed by many older adults, showing the kind of death they hoped for. Meanwhile, the sentence “hardships in old age being the real sufferings” reflected their fear of suffering before death, such as being paralysed in bed for years, being simultaneously having multiple severe symptoms, or being disliked, avoided, or even abandoned by children. *“I fear having a difficult time before death. I prefer to die now than endure this suffering.” (Participant 6)*

Despite this, many participants considered that their death (e.g., their time of death) and related sufferings were decided by their fate. Thus, worrying was seen as completely useless. What they could do at present was take one day at a time. *“The saying goes, ‘Your destiny was established when you were born and cried three times.’ Your life span and death have been determined since then. I don’t worry about my end, that day will come sooner or later. Now I just live one day at a time.” (Participant 9)*

## Discussion

To our knowledge, this study was the first qualitative study to explore the everyday life experiences related to living alone of community-dwelling older adults aged 80 years and over in China. The key challenges this oldest old population faced and associated strategies they used for coping were presented. The findings also provided new insights into understanding the difficulties and needs of this population.

The theme “difficulty in finding a sense of belonging” reflected the importance of belonging for those Chinese aged 80 years and beyond living alone. Their own home, their children’s home and others were ranked as three primary sources for acquiring a sense of belonging for our participants. This sequence of paths to acquire belonging was in line with the *guanxi* circles, a characteristic of Chinese social life that has a unique structure of “differential modes of association” such as family ties, familiar ties, and acquaintance ties (Luo & Yeh, 2012).

Influenced by Confucianism, family, the centre of everyday existence (Huang & Gove, 2012), is crucial for the Chinese because it provides affective feelings, security, and resources (Luo & Yeh, 2012). However, for older people who lived alone, especially those 80 years or older without regular visits from family or friends, their own household turned into “a lonely island” due to the lack of the presence of a trusted and intimate individual. Similar descriptions were reported in a study from South Korea, another East Asian country also influenced by Confucianism (Park & Cho, 1995; Yu et al., 2020). In contrast, the oldest old population who lived alone in rural areas of Norway and USA do not seem to feel lonely, but instead treasured the aloneness as solitude, felt comfortable in one’s own company, and found new meaning in life (Hinck, 2004; Ness et al., 2014a). The difference between the experiences of everyday experiences related to living alone at home could be attributed to cultural differences and population differences (urban versus rural) or caused by the varying level of inner strength of the participants (resilience) (Hinck, 2004; Ness et al., 2014a).

The subtheme “That is the home of my children” distinguished between their own home and their children’s home, indicating why the studied demographic preferred living alone at home. Most of the participants mentioned maintaining their autonomy, freedom, and maintaining their own lifestyle and dietary habits as the main cause. In addition, living autonomously at home was perceived positively as a privileged situation among those over 80 years of age in Switzerland (Bedin et al., 2019).

Another reason to live alone at home is to ensure respect, avoid conflict, and avoid becoming a burden to their children. Sexagenarians are usually healthier and more energetic. In China, they are more likely to live together with children to help their children’s family, such as taking care of grandchildren or doing housework (Ko & Hank, 2014). The oldest-old, in contrast, have little ability to assist their children in life and may need special attention or care because of slow reaction speeds, forgetfulness, and repeating things over and over. Under these circumstances, the oldest old individuals perceived a potential risk of being disrespected, seen as a burden, and being loathed by their children if they lived under the same roof for a certain time. Therefore, they chose not to live with their children. A similar phenomenon described as living separately to avoid conflicts and is considered better for the extended family was also mentioned among Hong Kong Chinese older adults (Lou & Ng, 2012).

In agreement with previous qualitative studies, visiting nearby places where older adults often gathered not only maintained social networks, reduced loneliness (Lou & Ng, 2012; Paine et al., 2022; Yu et al., 2020), but also enhanced overall self-esteem and collective well-being (Lou & Ng, 2012). However, our study further demonstrated the difficulties for some of the urban Chinese oldest old population to blend into social activities and maintain social relationships due to the possibility of being bullied by others.

The phenomenon of bullying in old age due to being perceived as slow was also shown in a study from Portugal (von Humboldt et al., 2022), which reflected the concept of ageism. Ageism is a complex domain, defined as discrimination, prejudice, or stereotype against people owing to their chronological age (Ayalon & Tesch-Römer, 2017). This phenomenon can occur at the individual level, in social networks, as well as an institutional and cultural level; and can be self-directed or other-directed ageism. All may have adverse effects on physical and mental health outcomes (Chang et al., 2020).

Some attention has been paid to ageism in the Chinese population, such as revealing the relationship between perceptions of older people as a burden and depression (Bai et al., 2016), reporting abuse (Liu & Hu, 2021). In addition, strategies aimed to reduce ageism have been proposed such as developing an intervention of “imagine that you were young” (Chen & Zhang, 2022) and advocating face-to-face intergenerational contact (Kwong & Yan, 2023). However, ageism in older adults living in the community remains an understudied area. Thus, more studies are encouraged to explore this area and targeted psychosocial interventions should be developed to address this issue.

The theme “striving to maintain independence” shows the importance of independence for the oldest old population and illustrates that maintaining independence is not an easy process. Financial independence is treated as an essential requirement to meet the living needs of participants. In an earlier phenomenological-hermeneutic study, financial independence (e.g., having their own house) had also been highlighted as the basis for acquiring and maintaining self-respect among Brazilian very old individuals (85 to 88 years old) (Caldas & Berterö, 2007). In the present study, participants used keeping property (e.g., house property, deposit) to achieve financial freedom and to acquire a sense of security (e.g., no need to request money from their children).

The adaptive strategies our participants adopted to meet basic living needs included using a cane or wheelchair while shopping in the store and doing necessary things on a daily basis when they felt the energy to do so. Similar strategies used by the oldest old rural adults in America were also reported (Hinck, 2004). Although we found that using technology to assist older adults seemed acceptable among the Chinese oldest old (e.g., online shopping), as the use of technology was influenced by various factors, such as psychological and contextual factors (Peek et al., 2016). Thus, further studies should investigate the perceptions, needs and experiences of using technology for assisting ageing in place among the Chinese community-dwelling oldest old individuals.

The subtheme “worries left untold” was a strategy to maintain independence and was an optimal choice after realizing the disadvantages were far beyond the advantages of disclosing bad news. Sharing bad information with children might damage their familiar lifestyle and cause conflict between the whole family, influencing family harmony. It is noteworthy that family harmony is a core element of Chinese family function, which uniquely contributes to family happiness (Lam et al., 2012). Most Chinese believe that “harmony in the family leads to prosperity in all undertakings” (Jiang, 2019). Therefore, the oldest old Chinese individuals in this study only reported good news when asked by children.

Identical to a Korean study (Yu et al., 2020), our participants stated religion as a way to improve resilience and acquire emotional support when facing difficulties in life. It is worth noting that one participant received psychological comfort because her offspring (children and grandchildren) were high achievers and had settled in a highly developed country. This made her feel satisfied and she thought that all the hardships she endured while living alone was worthwhile. This is a manifestation of parental sacrifice, a central feature in the Chinese concept of family, where parents subordinate or even sacrifice their personal interests and needs to nurture children and honour the family as a whole (Leung & Shek, 2011).

The theme “hard to gain a sense of control” presented two things that the oldest old had difficulty in controlling: an ageing body and unknown death. According to Shallcross et al. (2013), age was associated with increased acceptance, decreased anxiety, and improved emotional well-being. Surprisingly, even those adults aged 80 years or over still did not accept their ageing and declining body and experienced shame and self-loathing. This interesting finding was consistent with a survey from Greece (Mantzoukas et al., 2021) and a qualitative study from Brazil (Caldas & Berterö, 2007), expressing dissatisfaction and resentment over the loss of their health status while ageing. That phenomenon was related to self-directed ageism, one type of ageism that can be directed towards oneself (Ayalon & Tesch-Römer, 2017). As Mantzoukas et al. (2021) stated, physical function limitations and bodily deterioration because of the ageing process could cause shame among older people, resulting in triggering defensive responses and provoking sentiments such as hostility, isolation, and depression. Therefore, exploring why some very old people were significantly disturbed by self-directed ageism and how to reduce related negative impacts needs further research.

In the present study, most participants were willing to share attitudes and perceptions towards death. Consistently, they were not worried about death itself, but more held the attitude of “let death take its course,” which was shown in a recent qualitative study regarding the life-and-death attitudes among the elderly under Traditional Chinese Culture (Lei et al., 2022). This may be because they had reached longevity, and if they were to die, their death will be treated as a “white happy event,” meaning the death resulted from a natural cause such as ageing with a content life and no great life regrets (Xu, 2007). However, the oldest Chinese were concerned about how they would die, which was also the same concern of those 95 years and older from the United Kingdom (Fleming et al., 2016). A good death was defined as being painless and symptom-free (Fu & Glasdam, 2022). Our participants enriched the meaning of good death by expressing “a quick death is a blessing.” Here, the oldest old may experience pain or other symptoms in the short term. However, they still regarded this as a satisfying death, because, in a quick death, they would not experience the hardships they most fear, such as being bedridden for years, becoming a burden to their family, or being disliked and avoided by children. Interestingly, despite worrying about how they would die, we found that many participants thought that there was nothing else they could do because their death had been predetermined by fate. These thoughts were influenced by traditional Chinese cultures, such as Taoism’s statement of respecting life and accepting fate or Confucius’ view that “heaven controls a man’s fortune” (Wei, 2017), both unconsciously helping most Chinese treat unknown death and potential suffering peacefully.

### Study limitations

The study has some limitations. First, most of the participants were female; male adults may experience more challenges and difficulties, particularly when living alone (von Heideken Wågert et al., 2020). Future studies should involve more male participants or exclusively explore challenges and difficulties, or daily life experiences related to living alone of (very) old men in the community in China. Second, participants were recruited from two cities in Zhejiang Province, one of nation’s wealthiest regions of China. If exploring the everyday life experience related to living alone of the oldest old in rural areas or other regions of China, such as central and western parts where the general economic status is less than that in eastern China, different stories and voices may be heard, and different findings may be generated.

### Implications for research and practice

The theme “difficult to find a sense of belonging” and the subtheme “home being a lonely island” reflected the loneliness that Chinese community-dwelling oldest old who lived alone experienced. Several interventions might be used to reduce loneliness, such as increased activity and participation in discussion groups, skills courses, and contact with animals (O’Rourke et al., 2018). As there is no one-size-fits-all method to address older adults’ loneliness or social isolation, future research should develop tailored and context-based interventions to suit the needs of the oldest old individuals with different degrees of loneliness.

In terms of the potential risks of the oldest old living alone at home striving to maintain independence and leaving worries untold, community nurses should regularly measure functional capacity, risk of falls, and home safety assessment of the oldest old to quickly resolve any potential risks (Xu et al., 2020). For the oldest old who hide bad news, community nurses and social workers may function as a path for older persons to share their experiences, improve psychological well-being, and address issues if necessary.

## Conclusion

The oldest old Chinese individuals living alone in the community are faced with several challenges in their daily life. They presented emotional needs for a sense of belonging but were difficult to address. Faced with a declining body and death, they found it difficult to gain a sense of control. Some strategies have been used to maintain their independence. Future research should focus on loneliness reduction for the oldest old, the phenomenon of ageism in advanced age, and how to apply technology to assist ageing in place.
